# *COL1A2* p.Gly1066Val variant identified in a Han Chinese family with osteogenesis imperfecta type I

**DOI:** 10.1002/mgg3.619

**Published:** 2019-03-04

**Authors:** Mingyuan Wang, Yi Guo, Pengfei Rong, Hongbo Xu, Lina Gong, Hao Deng, Lamei Yuan

**Affiliations:** ^1^ Center for Experimental Medicine The Third Xiangya Hospital, Central South University Changsha China; ^2^ Department of Medical Information Information Security and Big Data Research Institute, Central South University Changsha China; ^3^ Department of Radiology The Third Xiangya Hospital, Central South University Changsha China; ^4^ Department of Neurology The Third Xiangya Hospital, Central South University Changsha China

**Keywords:** *COL1A2*, heterozygous variant, osteogenesis imperfecta, procollagen

## Abstract

**Background:**

Osteogenesis imperfecta (OI), a genetically determined connective tissue disorder, is characterized by increased bone fragility and reduced bone mass. Clinical presentation severity ranges from very mild types with nearly no fractures to intrauterine fractures and perinatal lethality. It can be accompanied by blue sclerae, dentinogenesis imperfecta (DI), hearing loss, muscle weakness, ligament laxity, and skin fragility. This study sought to identify pathogenic gene variants in a four‐generation Han Chinese family with OI type I.

**Methods:**

In order to unveil the molecular genetic factors underlying the disease phenotype, whole exome sequencing in a member, with OI type I, of a Han Chinese family from Hunan, China was performed. The variant identified by whole exome sequencing was further tested by Sanger sequencing in the family members.

**Results:**

A heterozygous missense variant (NM_000089.3: c.3197G>T; NP_000080.2: p.Gly1066Val) in the collagen type I alpha 2 chain gene (*COL1A2*) was identified in four patients. It co‐segregated with the disease in the family.

**Conclusion:**

The sequence variant may be a disease‐causing factor resulting in abnormal type I procollagen synthesis and leading to OI type I. This finding has significant implications for genetic counseling and clinical monitoring of high‐risk families and may be helpful for understanding pathogenic mechanism of OI and developing therapies.

## 1 INTRODUCTION

Osteogenesis imperfecta (OI), a genetically determined connective tissue disorder, is characterized by increased bone fragility and reduced bone mass (Marini et al., 2017; van Dijk et al., 2011). OI prevalence, at birth, is estimated at 3‐7 per 100,000 (Marini et al., 2017). Extra‐skeletal features include blue sclerae, dentinogenesis imperfecta (DI), hearing loss, muscle weakness, ligament laxity, skin fragility, pulmonary complications, and cardiovascular involvement (Becker et al., 2011; Marini et al., 2017). Phenotypical severity ranges from very mild types with nearly no fractures through variable skeletal deformities to intrauterine fractures and perinatal death (Becker et al., 2011; Marini et al., 2017; van Dijk et al., 2011).

In 1979, an OI “Sillence classification” was proposed and remains in use. It is based on clinical and genetic findings: Type I is classic, nondeforming OI characterized by blue sclerae; Type II is a perinatally lethal form; Type III is a progressively deforming form; Type IV is the common variable form with white sclerae (Sillence, Senn, & Danks, 1979). With the significant progress unmasking OI's genetic basis, the original “Sillence classification” has evolved using emerging genetic etiology along with distinctive clinical manifestations (Rauch & Glorieux, 2004). Presently, at least 18 OI types have been described with 17 pathogenic genes identified (Alanay et al., 2010; Becker et al., 2011; Cabral et al., 2007; Cho et al., 2012; Duran et al., 2015; Keller et al., 2018; Keupp et al., 2013; Lapunzina et al., 2010; Leal et al., 2018; Lindert et al., 2016; Martínez‐Glez et al., 2012; Mendoza‐Londono et al., 2015; Morello et al., 2006; Shaheen et al., 2012; Steiner, Adsit, & Basel, 2013; Takagi, Matsushita, Nishimura, & Hasegawa, 2014; van Dijk et al., 2009). Five types (I‐V) are inherited as an autosomal dominant trait with variable disease phenotypes. The rest may appear as autosomal recessive or X‐linked hereditary inheritance patterns. Approximately 77%‐90% patients had heterozygous alterations in the collagen type I alpha 1 chain gene (*COL1A1*, OMIM 120150) and the collagen type I alpha 2 chain gene (*COL1A2*, OMIM 120160), that encode pro‐α1 and pro‐α2 chains of type I procollagen respectively and are responsible for OI type I‐IV (Marini et al., 2017; Steiner et al., 2013). There are about three times as many OI patients with *COL1A1* variants than those with *COL1A2* variants (Zhytnik et al., 2017). More than a thousand *COL1A2* gene variants have been described in the OI variant database (https://oi.gene.le.ac.uk/) (Dalgleish, 1997; van Dijk et al., 2011). Yet its pathogenesis remains poorly understood. In this context, a missense variant (NM_000089.3: c.3197G>T; NP_000080.2: p.Gly1066Val) in the *COL1A2* gene was identified by using whole exome sequencing (WES) and Sanger sequencing in a Han Chinese family. It may be the genetic etiology for this OI family and have important implications for genetic monitoring.

## 2 MATERIALS AND METHODS

### 2.1 Participators and clinical evaluations

**Figure 1:**
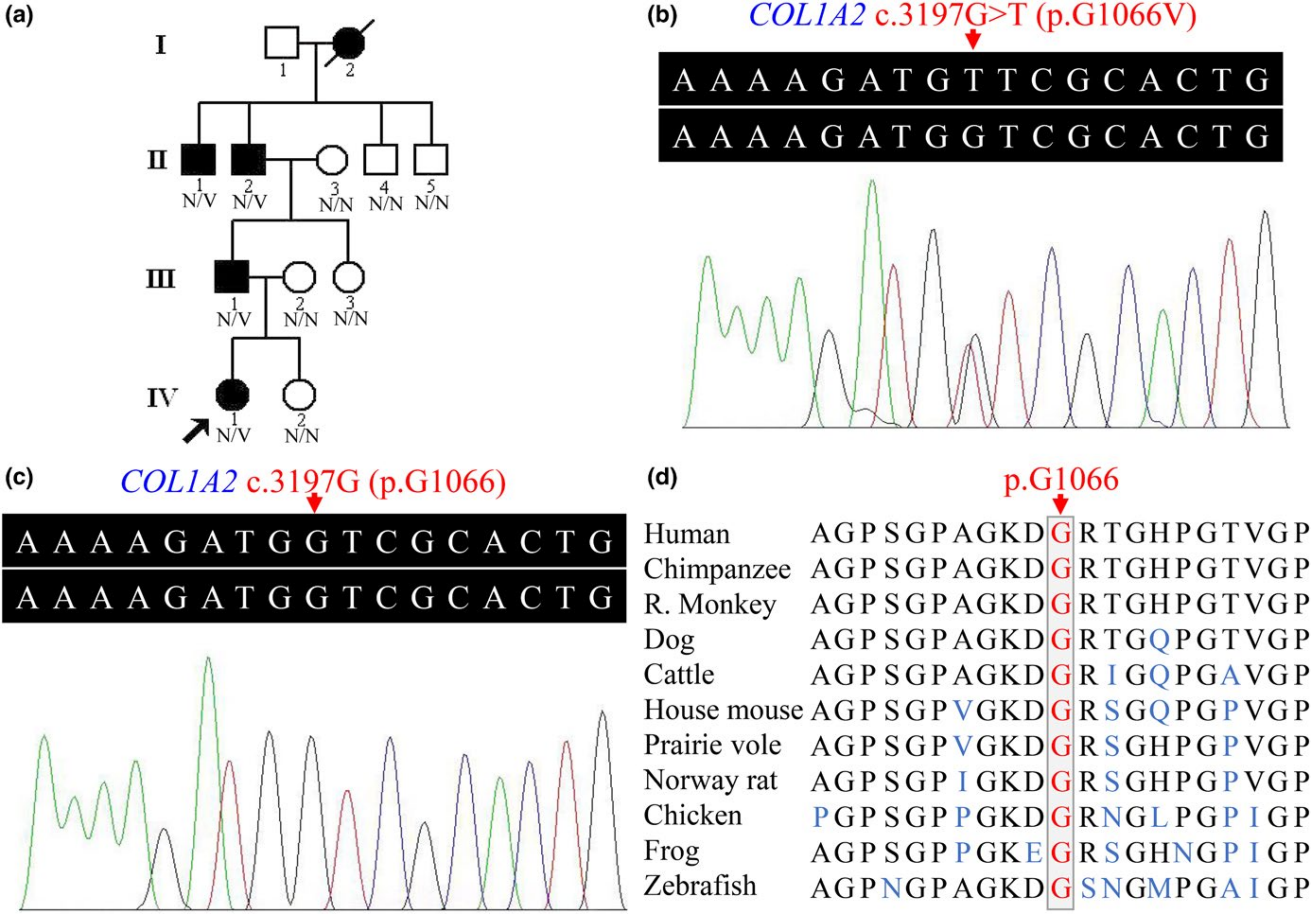
(a) Pedigree of the family with osteogenesis imperfecta showing affected cases (fully shaded). N: normal; V: the *COL1A2* c.3197G>T (p.Gly1066Val) variant. (b) The sequence with heterozygous *COL1A2* c.3197G>T variant of an affected individual (II:1). (c) The *COL1A2* gene sequence of a normal control (II:5). (d) Conservation analysis of the collagen type I pro‐α2 chain p.Gly1066 amino acid residue. *COL1A2*, the collagen type I alpha 2 chain gene

The subjects of this study belong to a four‐generation Han Chinese family with OI which comes from south central China (Figure 1a). Detailed clinical data and peripheral venous blood samples were obtained from 10 family members, including four individuals affected with OI (II:1, II:2, III:1, and IV:1) and six unaffected members (II:3, II:4, II:5, III:2, III:3, and IV:2). Clinical assessment and radiographic examinations were performed on the subjects of the family. Secondary osteoporosis and nonaccidental injuries were eliminated. The diagnostic process summarized by van Dijk et al. was employed (van Dijk et al., 2011). The research protocol was approved by the Institutional Review Board of the Third Xiangya Hospital, Central South University (Changsha, China), and adhered to Declaration of Helsinki tenets. All participants, or their guardians, executed written informed consent forms.

### 2.2 Exome capture

Genomic DNA (gDNA) was extracted from peripheral blood samples using standard procedures (Yuan et al., 2015). WES was performed on the proband (IV:1) by BGI‐Shenzhen, as previously described (Fan et al., 2019). Sequencing library construction was accomplished via a qualified gDNA sample randomly broken by sonication using Covaris E220 (Covaris, Brighton, UK), which yielded 150 to 250 bp of fragments. End‐repairing, phosphorylation, and A‐tailing reactions of the fragments were then conducted, and a ligation‐mediated, polymerase chain reaction amplification was followed. They were further modified, amplified, purified, and hybridized to the exome array for enrichment. Using the circular single stranded libraries, DNA nanoballs were formed via rolling circle amplification, and then loaded onto sequencing flow cells. The enriched DNA library underwent high‐throughput sequencing according to the BGISEQ‐500 protocol (Huang et al., 2017).

### 2.3 Variant analysis

Raw data from the BGISEQ machine was filtered to generate clean data. This was aligned to the human reference genome sequence (GRCh37/hg19) from the UCSC Genome Browser by using the Burrows–Wheeler Aligner (BWA) software program (Xia et al., 2015). Local realignment around insertions‐deletions (InDels) and base quality score recalibrations were performed using the Genome Analysis Toolkit (GATK, https://www.broadinstitute.org/gatk/guide/best-practices). Picard tools (http://broadinstitute.github.io/picard/) removed duplicate reads. The SnpEff tool (http://snpeff.sourceforge.net/SnpEff_manual.html) annotated variants including single nucleotide polymorphisms (SNPs) and InDels, as previously described (Xiao et al., 2018).

All candidate variants were filtered against several public databases including: the 1000 Genomes Project (http://www.internationalgenome.org/), the SNP database (dbSNP, https://www.ncbi.nlm.nih.gov/snp), and the NHLBI exome sequencing project (ESP) 6500 database, as well as the in‐house BGI exome database. Online tools, including Polymorphism Phenotyping version 2 (PolyPhen‐2, http://genetics.bwh.harvard.edu/pph2/index.shtml), Sorting Intolerant from Tolerant (SIFT, http://sift.jcvi.org/), MutationAssessor (MA, http://mutationassessor.org/), Condel and Functional Analysis through Hidden Markov Models (FATHMM, http://fathmm.biocompute.org.uk/), were used to predict the possible impacts of amino acid substitutions. Sanger sequencing was employed to verify the identified potential disease‐causing variant with an ABI3500 sequencer (Applied Biosystems Inc., Foster City, CA) (Xiao et al., 2018; Zheng et al., 2016). Primer sequences designed by Primer3 (http://primer3.ut.ee/) were as follows: 5′‐AGGCTAAAGCGAGCAGTGAG‐3′ and 5′‐AAAACATTCCTTAGGTCCGTGA‐3′. GenBank NG_007405.1 was adopted as the reference sequence. MutationTaster (http://www.mutationtaster.org/) evaluated the possible impact of amino acid substitution, as previously described (Hu et al., 2017). Basic Local Alignment Search Tool (BLAST, https://blast.st-va.ncbi.nlm.nih.gov/Blast.cgi) was used for multiple protein sequence alignments.

## 3 RESULTS

### 3.1 Clinical characteristics of the pedigree

**Figure 2:**
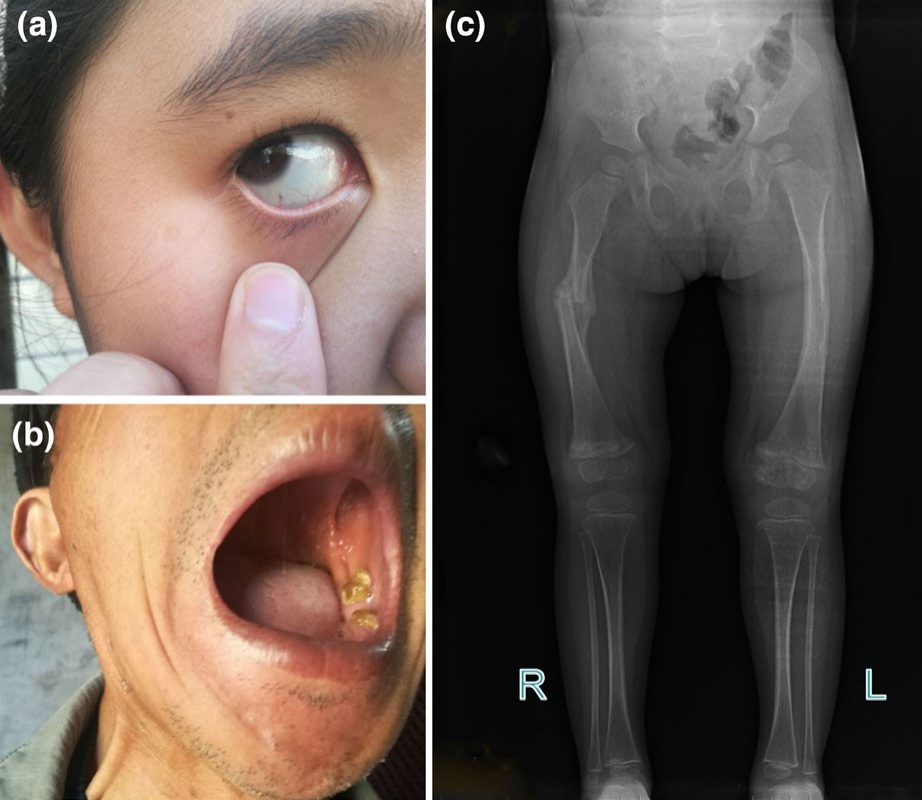
(a) The proband (IV:1) presents with blue sclera. (b) Clinical picture shows dentinogenesis imperfecta in patient (II:2). (c) Radiograph shows fractures and abnormal callus formation of the proband (IV:1) resulting in slight deformations of long bones

The affected subjects (II:1, II:2, III:1, and IV:1) had similar clinical abnormalities and had been diagnosed based on symptoms (Figure 2) by osteologists from the Third Xiangya Hospital, Central South University. Family members denied consanguineous marriages. Patient IV:1 was an 11‐year‐old girl with blue sclerae (Figure 2a), who had suffered a right femoral fracture at age 1. She gradually developed multiple fractures. Imaging data showed multiple fractures and abnormal callus formation on the right femur and a slight deformation of the left femur (Figure 2c). All patients (II:1, II:2, III:1 and IV:1) presented with blue sclerae, DI, and multiple bone fractures resulting from minimal trauma. The clinical features of the pedigree are summarized in Table 1.

**Table 1:** Clinical and genetic characteristics of family members with *COL1A2 *c.3197G>T variant

Subject	II:1	II:2	III:1	IV:1
Sex	Male	Male	Male	Female
Zygosity	Heterozygous	Heterozygous	Heterozygous	Heterozygous
Ethnic background	Han Chinese	Han Chinese	Han Chinese	Han Chinese
Age (years)	66	62	35	11
Height (centimeter)	160	155	169	146
Weight (kilogram)	55.6	58.5	82.4	50.0
Fractures	Multiple	Multiple	Multiple	Multiple
Sclerae	Pale blue	Pale blue	Pale blue	Blue
Hearing loss	No	No	No	No
Dentinogenesis imperfecta	Yes	Yes	Yes	Yes
Bone deformity	Unknown	Unknown	Unknown	Moderate
Stature	Normal	Normal	Normal	Normal
Clinical presentation severity	Moderate	Moderate	Moderate	Severe

Note

*COL1A2*, the collagen type I alpha 2 chain gene.

### 3.2 Whole exome sequencing

There were 235.53 million clean reads and 210.56 million total effective reads generated, with 99.93% aligned to the human reference genome. The mean sequencing depth was 253.04. The fraction of bases covered by the target sequence at more than 10× was 99.65%. A total of 103,343 SNPs and 18,066 InDels were detected.

### 3.3 *COL1A2* mutation screening

A prioritization scheme was carried out to identify the pathogenic variant (Wu et al., 2016). Variants in the 1,000 Genomes Project, dbSNP and NHLBI ESP6500 with a minor allele frequency of ≥1% were removed. Variants were defined as deleterious via bioinformatics tools. A heterozygous missense variant (NM_000089.3: c.3197G>T; NP_000080.2: p.Gly1066Val) in *COL1A2* exon 48 was found to be the cause for OI in the proband (IV:1). It was absent from 2,375 Chinese controls in the in‐house BGI exome database. Using Sanger sequencing, the heterozygous variant, c.3197G>T, in the *COL1A2 *gene, was confirmed. It was found in three other affected subjects (II:1, II:2, and III:1, Figure 1b), but absent from six unaffected family members (II:3, II:4, II:5, III:2, III:3, and IV:2, Figure 1c). MutationTaster software analysis revealed that the *COL1A2* c.3197G>T variant could be a disease‐causing variant with a probability value close to 1, indicating it is highly secure. The glycine at position 1066 (p.G1066) is highly conserved across vertebrates, from human to zebrafish (Figure 1d).

## 4 DISCUSSION

OI is a rare bone disorder characterized chiefly by bone brittleness and a tendency to fracture. Mutations in *COL1A1/2* genes, which encode the pro‐α1 and pro‐α2 chains of type I procollagen, were reportedly responsible for most OI (Martin & Shapiro, 2007). Given that OI is highly heterogeneous and the causative *COL1A1/2* genes are large (Alanay et al., 2010; Steiner et al., 2013), large‐scale OI‐related variant analyses using ordinary Sanger sequencing are time‐consuming and cost‐expensive. WES is a currently available effective approach for screening pathogenic variants of OI (Keller et al., 2018; Mackenroth et al., 2016). A heterozygous variant (NM_000089.3: c.3197G>T; NP_000080.2: p.Gly1066Val) in the *COL1A2 *gene affecting the helical region was identified in this Han Chinese family with OI. Four affected subjects (II:1, II:2, III:1, and IV:1) carried the heterozygous *COL1A2* c.3197G>T variant. Six unaffected family members were free of c.3197G>T variant. These facts suggest that the *COL1A2* c.3197G>T variant co‐segregates strongly with the OI phenotype. Clinical manifestations including fracture frequency, sclerae color, DI, bone deformity, and severity varied among the four affected subjects. The proband manifested the strongest symptoms (Table 1). Background genes interference and factors such as epigenetics and the environment might contribute to this family's variety of OI type I phenotypes. This variant was previously reported in a 5‐year‐old Chinese male with OI type I, suffering multiple fractures and extra‐skeletal manifestations of blue sclerae and brittle teeth (Wang et al., 2015). This variant in two independent families suggests that it may have a founder effect in Chinese, or it is a recurrent variant.

OI type I, unlike other OI types, rarely presents with neonatal fractures. This tendency is constant during childhood and puberty, and decreases thereafter. It often increases following menopause and in men over 50. With adequate orthopedic care, fractures may heal rapidly without deformity (Steiner et al., 2013). Additional clinical features may be blue sclerae, late‐onset hearing loss and joint laxity, with no apparent short stature, common DI, or bone deformity (Marini et al., 2017; Steiner et al., 2013; van Dijk et al., 2011). The OI type I diagnosis of this family was based on clinical and genetic testing.

The *COL1A2* gene, mapped to chromosome 7q21.3, spans approximately 37 kb and comprises 52 exons. It encodes the pro‐α2 chain of type I collagen, which is a protein observed in most connective tissues and widely distributed in the extracellular matrix of bone, skin, ligament, and tendon (Dalgleish, 1997; Marini et al., 2017; Wang et al., 2015). There are 1,051 variants, including 988 substitutions, 40 deletions, 17 duplications, and six InDels in the *COL1A2* gene, which have been recorded in the OI variant database (https://oi.gene.le.ac.uk/, updated on 28 November 2018). The common *COL1A2 *variants lead to glycine substitutions within the pro‐α2 chain triple helical domain. The pro‐α2 chain major structure is a triple helical segment formed by multiple Gly‐X‐Y triplet repeat units. Glycine is the only residue tiny enough to allow proper chain folding. A study of 291 independent *COL1A2* variants that resulted in glycine substitution found that 81.1% of *COL1A2* variants are nonlethal, and that about 13.9% of the alterations are valine (Marini et al., 2007). In this study, hydrophobic glycine was substituted by hydrophobic valine (p.Gly1066Val), which is an α‐amino acid with a branched nonpolar side chain. Two α1 chains and one α2 chain form the type I collagen triple helix, whose propagation may be delayed when glycine is substituted, and all three chains are liable to have post‐translational overmodification. Some assembled trimers resulted from substitutions are never secreted (Marini et al., 2007; Steiner et al., 2013). Variants can result in a reduced amount of procollagen secretion and abnormal structure of protein in the matrix (Alanay et al., 2010; Steiner et al., 2013). The variant present in the subjects of this study appears to be nonlethal, which is consistent with the finding that *COL1A2* variants are predominantly nonlethal, and the substitutions of glycine by hydrophobic amino acids are more likely to be nonlethal variations compared with those by hydrophilic amino acids (Marini et al., 2007).

Treatments of primary and secondary OI complications include pharmacological management, orthopedics, physiotherapy, and dental or hearing therapies (van Dijk et al., 2011). In *Col1a2^+/p.G610C^* mice, the secretion and bone matrix incorporation of defective α2(I) chain in ~50% of type I collagen heterotrimers result in bone mass and strength reductions (Masci et al., 2016). Combining anti‐sclerostin antibody and zoledronic acid has been reported as increases in tissue mineral density and cortical thickness, and sheds light on OI therapies (Little et al., 2017). Using adeno‐associated virus vectors which disrupt mutated *COL1A2* genes in OI mesenchymal stem cells has resulted in normal type I procollagen and bone generation, which may be another promising therapeutic technology for OI (Chamberlain et al., 2008).

The discovery of this *COL1A2* c.3197G>T variant may assist in genetic counseling, embryonic screening of *in vitro* fertilized embryos and prenatal genetic diagnosis. This could reduce familial transmission in this Han Chinese family and contribute to potential gene‐targeted therapies.

In conclusion, a c.3197G>T (p.Gly1066Val) transversion was identified in a Han Chinese family with OI type I sufferers. Further studies may contribute to improved clinical care, genetic screening and counseling, while facilitating effective OI treatment.

## CONFLICT OF INTEREST

All authors declare that they have no conflict of interest.
